# Accidental Section of the Ureter during Ovariotomy

**Published:** 1877-08

**Authors:** 


### Accidental Section of the Ureter during Ovariotomy.

———

Dr. Nussbaum gives some interesting details of this grave accident in the *Aertlichs Intelligen. Halt*, 1876, and *Saget Meldon*, May 25th, 1877. He recalls the case of Simon of Heidelburg, who, in 1867, during an operation, destroyed part of the left ureter, and at the same time a part of the uterus. The urine escaped by the uterine orifice and the vagina, at the same time, by an abdominal fistula at the top of the incision.

Dr. Nussbaum's case is different. In this patient (who was the ninety-sixth on whom he performed ovariotomy) he found a considerable quantity of pus and urine, after making the abdominal incision. After recovery, there still remained an abdominal urinary fistula, to remedy which Dr. Nussbaum had three courses opened to him.

1. To obliterate the ureter, with the hope of obtaining atrophy of the corresponding kidney.
2. To form an artificial ureter.
3. To excise the kidney.

He decided on the second method, and, having chloroformed the patient, he incised the urethra, and penetrated into the vagina with a trocar carrying a drainage tube; and, having perforated the vesical walls, the tube was introduced into the artificial cavity where the urine accumulated. The drain was twenty centimetres in length, and opened out at the meatus urinarius. We must admire the wonderful recuperative power of Nature, especially towards the gentler sex; for in this case, we are informed, the artificial apparatus succeeded. The urine secreted and passed by the new ureter, the abdominal fistula healed, and the patient completely recovered.—*London Medical Press.*

